# Microstructures and Mechanical Properties of Al-Zn-Mg-Cu Alloys under Multi-Directional Severe Strain and Aging

**DOI:** 10.3390/ma16124441

**Published:** 2023-06-17

**Authors:** Chunhua Wei, Zhixin Lei, Sijie Du, Rongyou Chen, Yutang Yin, Chenglin Niu, Zhengbing Xu

**Affiliations:** 1 MOE Key Laboratory of New Processing Technology for Non-Ferrous Metals and Materials, Guangxi Key Laboratory of Processing for Non-Ferrous Metals and Featured Materials, Center of Ecological Collaborative Innovation for Aluminium Industry in Guangxi, Guangxi University, Nanning 530004, China; 2 School of Resources, Environment and Materials, Guangxi University, Nanning 530004, China

**Keywords:** Al-Zn-Mg-Cu alloys, multiaxial forging, aging, precipitation, hardness

## Abstract

Microstructure is a significant factor that influences the mechanical properties of alloys. The effect of multiaxial forging (MAF) and subsequent aging treatment on the precipitated phases of Al-Zn-Mg-Cu alloy remains unclear. Therefore, an Al-Zn-Mg-Cu alloy was processed by means of solid solution and aging treatment, and MAF and aging treatment in this work, and the composition and distribution of precipitated phases were characterized in detail. The MAF results for dislocation multiplication and grain refinement were found. The high density of dislocation greatly accelerates the nucleation and growth of precipitated phases. Thus, the GP-zones almost transform into precipitated phases during subsequent aging. The MAF and aging alloy has more precipitated phases than the solid solution and aging treated alloy. The precipitates on the grain boundary are coarse and discontinuously distributed due to dislocation and grain boundary promoting the nucleation, growth and coarsening of the precipitates. The hardness, strength, ductility and microstructures of the alloy have been studied. Without compromising the ductility much, the MAF and aging alloy has higher hardness and strength, with values of 202 HV and 606 MPa, respectively, and an appreciable ductility of 16.2%.

## 1. Introduction

Al-Zn-Mg-Cu alloys have been widely used in aerospace applications as important structural materials due to their high specific strength, ductility, toughness and good processability [1,2]. In the conventional process, these alloys achieve high strength by means of fine-scale precipitation. However, it is difficult to simultaneously achieve high strength, ductility, fracture toughness and stress corrosion cracking resistance of Al-Zn-Mg-Cu alloys [3,4,5,6]. Some researchers, such as Bhat et al. [7,8] and Thangaraj et al. [9], improved the corrosion resistance of the layer formed on the mild steel surface by optimizing the coating preparation process. These studies are innovative and interesting, as the process maintains the basic mechanical properties of the matrix while enhancing corrosion resistance.

Some investigations have been carried out on the aging precipitation behavior of Al-Zn-Mg-Cu alloys, which show that the usual precipitation sequences may be summarized as [10,11,12]: solid solution-GP zone-metastable η’ phase-stable η phase (MgZn_2_). Moreover, the size and distribution of precipitates in Al-Zn-Mg-Cu alloys are significant factors that influence the mechanical properties and corrosion resistance. For example, Han and co-workers [13] reported that when increasing the pre-stretching, the η phase in 7050 aluminum alloy become coarser, and the η’ phase is reduced after double-stage aging, which leads to a decrease in strength and increase in toughness. Cai and co-workers [14] studied the effect of pre-deformation on the aging precipitation behavior of 7050 aluminum alloy, and the results showed that pre-deformation accelerates both the formation and spheroidization of fine precipitates, and the alloy displays superior strength and elongation.

It is worth noting that severe plastic deformation (SPD) is an effective way to achieve materials with ultrafine-grained structure and high strength [15]. The SPD processing in precipitation-strengthened aluminium alloys has attracted a growing interest from specialists in materials science, with the aim of keeping mechanical properties and corrosion resistance simultaneously at a high level [16,17,18,19,20,21]. Huang et al. [22] processed an Al-Zn-Mg-Cu alloy using SPD and aging treatment. This resulted in a refined microstructure, with coarse and discontinuous distribution of grain boundary precipitates. The ductility and strength simultaneously increased to higher values, and the fracture toughness also improved. Aoba et al. [23] compared and analyzed the influence of natural aging and artificial aging on the mechanical properties of Al-Zn-Mg-Cu alloy processed by SPD. The results showed that the yield strength was increased by 96 MPa due to SPD processing, and the mechanical properties and microstructure were stably maintained during aging while achieving further improvement in mechanical properties. However, due to the introduction of severe strain that leads to dislocation multiplication, it is necessary to explore further and clarify the microstructure evolution, especially of the precipitation characteristics during aging, which makes this an interesting SPD and heat treatment process to study.

Multiaxial forging (MAF) is one of many SPD processes used for preparing bulk materials, which is often applied for engineering purposes [15]. In this work, an Al-Zn-Mg-Cu alloy was processed by means of MAF and aging treatment. The microstructure was examined and its mechanical properties were studied. Based on this, the effect of MAF and aging treatment on the microstructure and mechanical properties of an Al-Zn-Mg-Cu alloy will be discussed in detail. This study is expected to provide some theoretical references for the design of Al-Zn-Mg-Cu alloys for use in complex environments.

## 2. Materials and Methods

A hot-extruded AA7075 aluminium alloy rod with a cross-section of 30 mm × 60 mm was used in the present experiment. It contained alloy elements of 5.72 Zn, 2.40 Mg, 1.80 Cu, 0.15 Fe, 0.05 Si, 0.05 Mn, 0.02 Cr, 0.06 Ti and balanced Al (all in weight percentage). The samples were cut into cubes with dimensions of 15 mm × 15 mm × 15 mm and the solution was treated at 470 °C for 2 h, then quenched in water (solution-treated sample). Then, using a hydraulic machine, the solution-treated sample was processed alternately by changing the loading direction by 90° (i.e., x-y-z-x) at room temperature (MAF sample). The forging was two cyclic and carried out at a strain rate of about 10 s^−1^. Every stage of forging produced about 10% strain, and the total true strain ΣΔ*ε* was about 60%. Then, the MAF samples was aged at 120 °C for 4 h, 8 h, 12 h, 16 h, 20 h (MAF-A20 sample), 24 h and 28 h, respectively. Generally, the AA7075 aluminium alloy treated with T6 has considerable strength and ductility. Therefore, the sample used for comparison was heated to 470 °C for 2 h and subsequently quenched in water, then aged at 120 °C for 24 h (T6-treated sample). The details of the preparation technology used for test samples are listed in Table 1.

The phase evolution of the samples was identified by XRD with Cu Kα in a Rigaku D/MAX 2500V X-ray diffractometer (RIGAKU, Tokyo, Japan). The microstructure of samples was examined by a FEI Tecnai G2 F20 transmission electron microscope (TEM, operating at a voltage of 200 kV) (FEI, Hillsboro, OR, USA). TEM samples were prepared by mechanical grinding and then thinned by ion thinning at low temperature. The fracture morphology of tensile samples was observed by a S-3400 scanning electron microscope (SEM) (HITACHI, Tokyo, Japan).

An AVH-5L microhardness tester (Beijing Time Technologies Co., Ltd., Beijing, China) was used to measure the hardness of samples under a load of 0.29 N for 15 s. The surface for the hardness measurement was a plane perpendicular to the rolling direction of the sample, and seven positions were randomly measured on the surface, with the average value taken. According to the standard GB/T 228.1-2010 [24], tensile specimens were machined from the T6-treated, MAF and the MAF-A20 billets, with a gauge size of 5 mm length, 1 mm width and 1 mm thickness. The tensile properties were determined on an Instron 8801 Materials testing machine (INSTRON, High Wycombe, UK) at a constant rate of 1 mm/min. A minimum of three good reproducible tests were performed on each type of sample.

## 3. Results and Discussions

**Figure 1:**
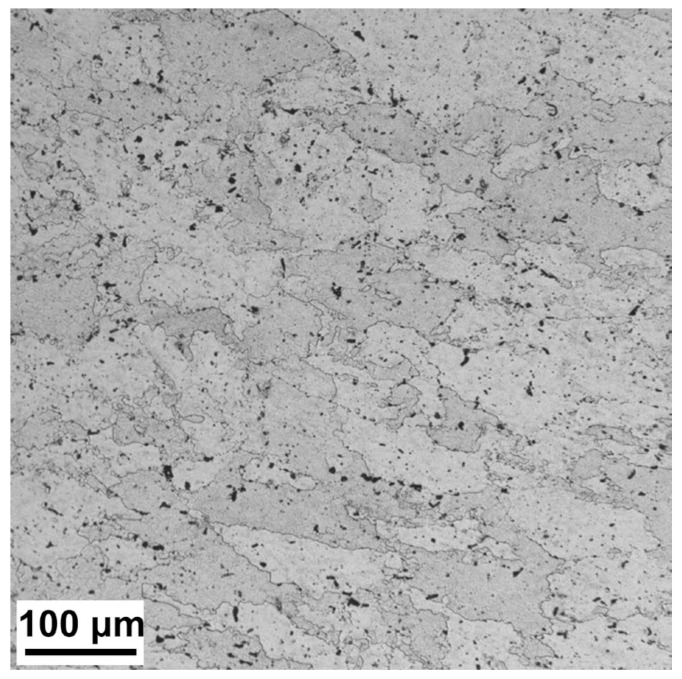
Cross-sectional optical microstructure of the T6-treated sample.

**Figure 2:**
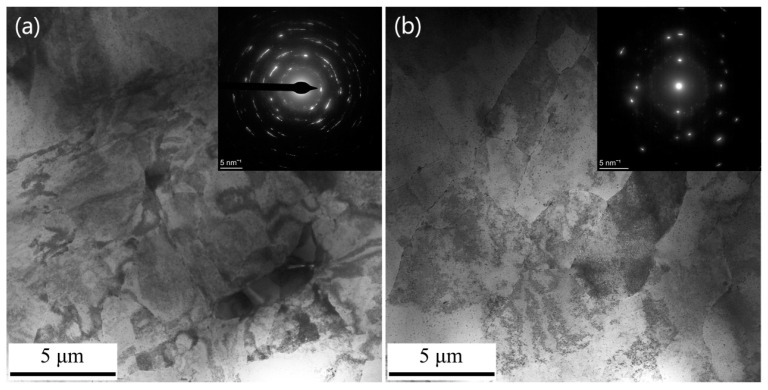
Cross-sectional TEM image of the MAF sample (**a**) and the MAF-A20 sample (**b**).

Figure 1 shows a cross-sectional optical microscopic microstructure of the T6-treated sample. It can be seen that the microstructure of the sample is coarse-grained. Figure 2 shows a cross-sectional TEM microstructure of the MAF sample and the MAF-A20 sample. It can be seen that there is a typical SPD microstructure with high dislocation density and dislocation tangles, as exhibited in Figure 2a. The selected area electron diffraction (SAED) pattern shows diffusive diffraction spots, which suggested small misorientations among the fine grains. The SPD microstructure with high dislocation density can still be found in the MAF-A20 sample, as shown in Figure 2b.

**Figure 3:**
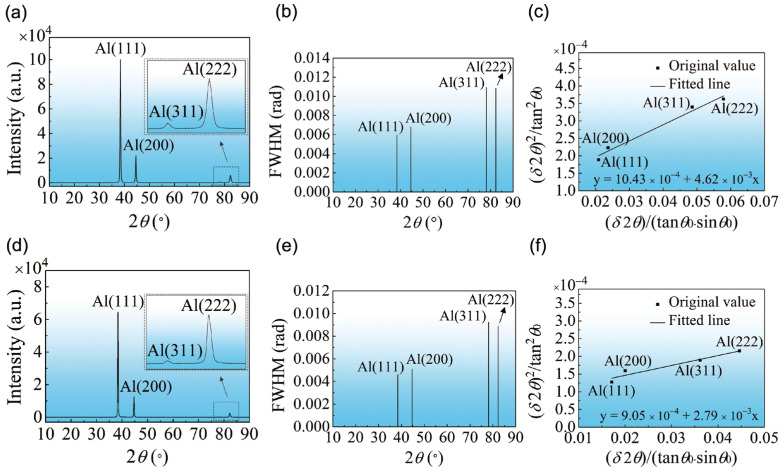
XRD data analysis results of the MAF sample (**a**–**c**) and the MAF-A20 sample (**d**–**f**): (**a**,**d**) XRD pattern of low-rate scanning, (**b**,**e**) FWHM values of each diffraction peak, and (**c**,**f**) the plots of ${\left(\delta 2\theta \right)}^{2}/tan^{2}\theta_{0}-\delta 2\theta /tan\theta_{0}sin\theta_{0}$ fitted linearly.

Figure 3 shows the XRD data analysis results of the MAF sample and the MAF-A20 sample. As shown in Figure 3a,d, all detectable peaks can be represented by the *α*-Al structure according to standard reference data. The refinement of grain size and the introduction of lattice strain is the main reason for the XRD peak broadening [25].

According to Hall’s method, the following formula can be written as [25]:

(1) $$\frac{{\left(\delta 2\theta \right)}^{2}}{tan^{2}\theta_{0}}=25\langle e^{2}\rangle +\frac{\lambda }{d}\left(\frac{\delta 2\theta }{tan\theta_{0}sin\theta_{0}}\right)$$

where *δ*2*θ* is the integral breadth (which is equal to FWHM/0.9) [26,27], *θ*_0_ is the Bragg angle corresponding to *δ*2*θ*, *e* is the lattice strain, *λ* is the wavelength of Cu Kα radiation (0.154 nm), and *d* is the crystallite size.

Figure 3b,e shows the FWHM values of each diffraction peak obtained by Gaussian fitting. The linear fitting result of ${\left(\delta 2\theta \right)}^{2}/tan^{2}\theta_{0}-\delta 2\theta /tan\theta_{0}sin\theta_{0}$ is shown in Figure 3c,f. The crystallite size (*d*) and the root mean square lattice strain (<*e*^2^>^1/2^) are calculated according to the slope and intercept of the linear fitting result. Finally, the dislocation density is given by the following Equation (2) [20]:

(2) $$\rho =2\sqrt{3}{\langle e^{2}\rangle }^{1/2}/\left(db\right)$$

where *b* is a Burgers vector (0.286 nm) [16]. Therefore, the dislocation density of the MAF sample is 7.4 × 10^14^/m^2^, which is a relatively high value. Due to the recovery, the dislocation density of the MAF-A20 sample slightly decreased but still remained at 4.2 × 10^14^/m^2^. All computing parameters and the calculated dislocation density for the MAF sample and MAF-A20 sample are shown in Table 2.

**Figure 4:**
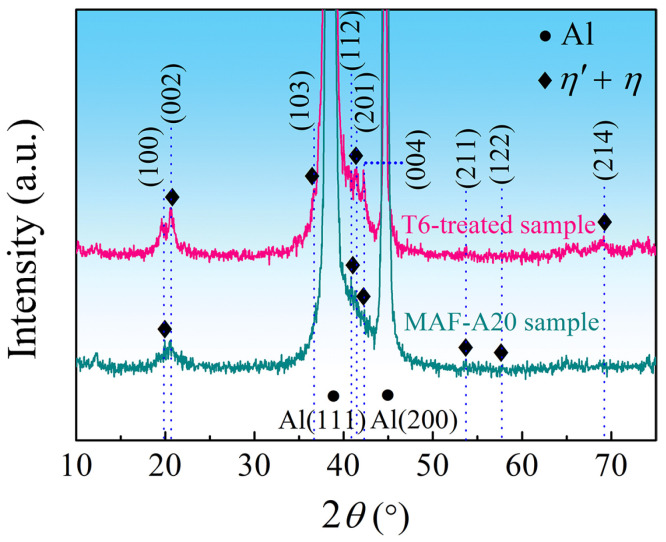
XRD patterns showing the η’ and η phases in the T6-treated and MAF-A20 samples. The blue dashed lines indicate the 2*θ* positions corresponding to each diffraction peak.

**Figure 5:**
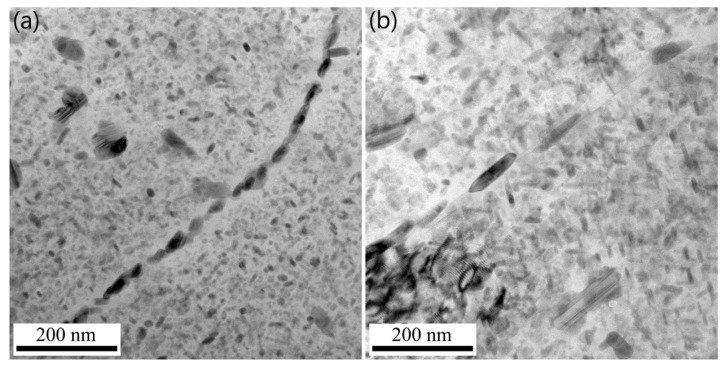
TEM images of grain boundary precipitates in the samples: (**a**) T6-treated; (**b**) MAF-A20.

XRD patterns of the T6-treated and the MAF-A20 samples are presented in Figure 4. It can be ascertained that the precipitates are η’ and η phases. Figure 5 shows the grain boundary precipitates in the samples. For the T6-treated sample, the precipitates on the grain boundary are small and continuously distributed, as presented in Figure 5a. However, for the MAF-A20 sample, the grain boundaries are decorated with the coarse and discontinuous precipitates, as shown in Figure 5b.

**Figure 6:**
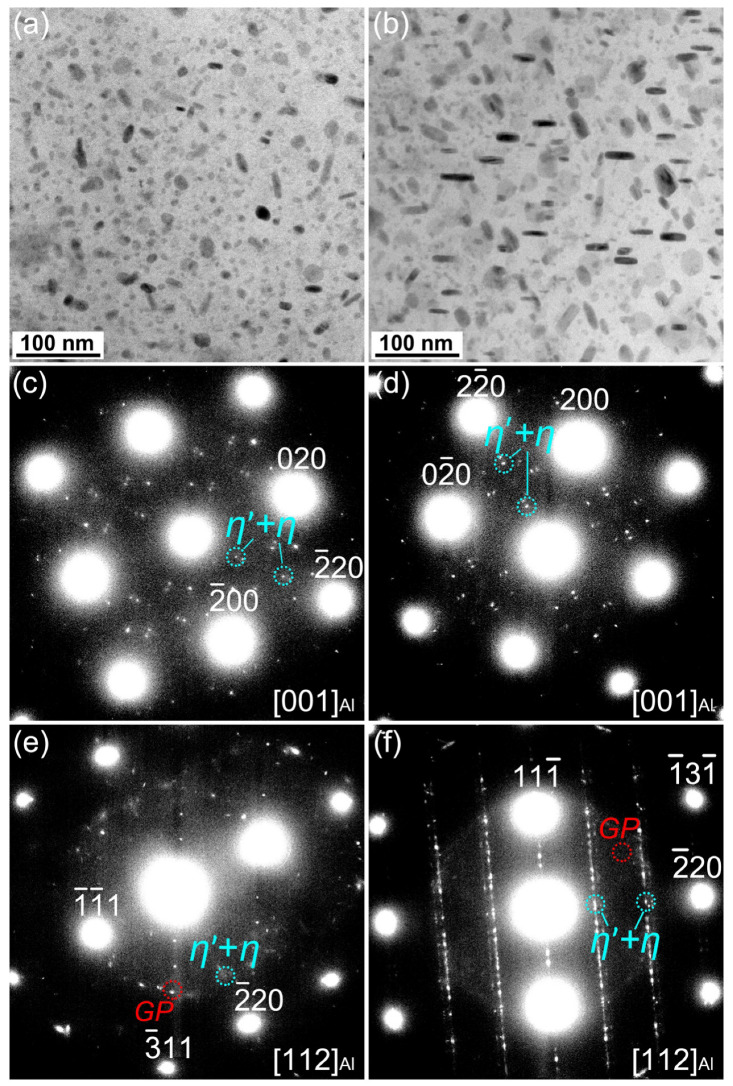
TEM images and corresponding SAED patterns of precipitates within grains of the T6-treated sample (**a**,**c**,**e**) and the MAF-A20 sample (**b**,**d**,**f**).

Figure 6 shows the TEM images of precipitates within the grains and SAED patterns of the T6-treated and MAF-A20 samples. From Figure 6a,b, it can be seen that the precipitates are distributed homogeneously within the grains. As shown in Figure 6c,e, the diffraction spots of η’ and η phases in the T6-treated sample are weaker, while the diffraction spots of GP-zones are stronger. According to the precipitation sequences of aluminium alloy, the precipitated products in the T6-treated sample are mainly GP-zones and a small amount of fine η’ phase. In Figure 6d,f, the GP-zones have almost disappeared, and the diffraction spots of η’ and η phases are stronger than those of the T6-treated sample. This indicates that the GP-zones almost completely transformed into η’ phase and a small amount of larger η phase in the MAF-A20 sample.

**Figure 7:**
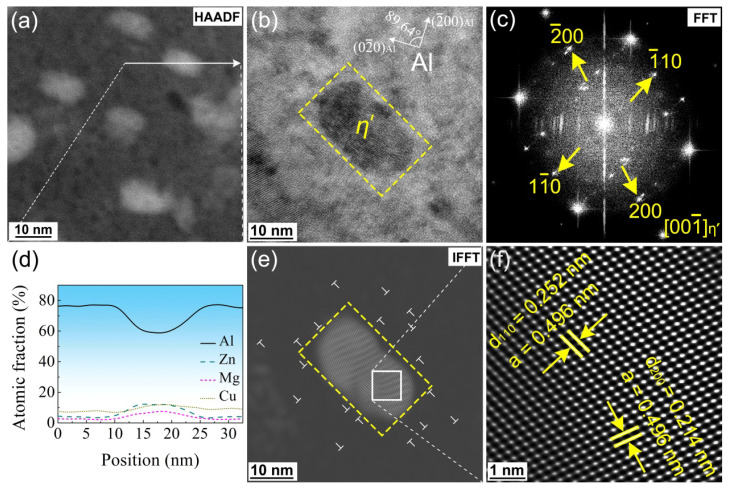
TEM images of the η’ phase (marked by a yellow dashed box) of the MAF-A20 sample: (**a**) HAADF-STEM, (**b**) HRTEM, (**c**) FFT, (**d**) EDS-Line Scan result of the white arrow from (**a**), (**e**) IFFT, (**f**) further enlargement view of the white box from (**e**).

**Figure 8:**
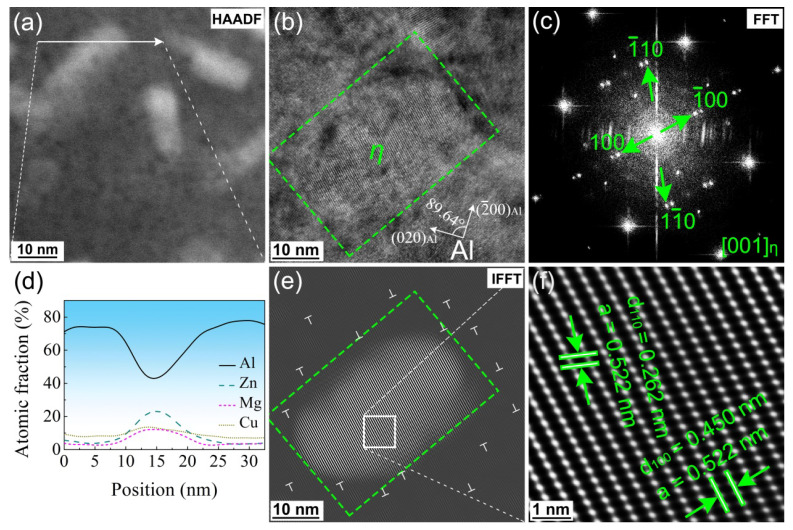
TEM images of the η phase (marked by a green dashed box) of the MAF-A20 sample: (**a**) HAADF-STEM, (**b**) HRTEM, (**c**) FFT, (**d**) EDS-Line Scan result of the white arrow from (**a**), (**e**) IFFT, (**f**) further enlargement view of the white box from (**e**).

Figure 7 and Figure 8 characterize the η’ phase (a = 0.496 nm, c = 1.402 nm) and the η phase (MgZn_2_, a = 0.522 nm, c = 0.857 nm) in the MAF-A20 sample, respectively. Figure 7a and Figure 8a shows the high-angle annular dark-field scanning transmission electron microscopy (HAADF-STEM) image of the η’ phase and η phase. As shown in Figure 7d and Figure 8d, from the energy dispersive X-ray spectroscopy-line scan (EDS-Line Scan), a η’ phase with an atomic ratio of Zn/Mg = ~1.5:1 and η phase with an atomic ratio of Zn/Mg = 2:1 can be easily estimated. Figure 7b and Figure 8b show the high-resolution transmission electron microscopy (HRTEM) image of the η’ phase (semi-coherent, plate-shaped, and metastable hexagonal, with a size of approximately 10–30 nm) and η phase (non-coherent, equiaxed, and stable hexagonal, with a size of approximately 30–50 nm), respectively. As shown in Figure 7c and Figure 8c, from the fast Fourier transform (FFT) pattern, the information of crystal plane corresponding to the precipitates has been marked. In the inverse fast Fourier transform (IFFT) pattern of Figure 7e and Figure 8e, it can be verified that the η’ and η phases is incoherent with the Al matrix. In addition, the lattice distortion is caused by a great number of dislocations gathered around the precipitates, which suggests that the precipitates have a hindering effect on the dislocations.

**Figure 9:**
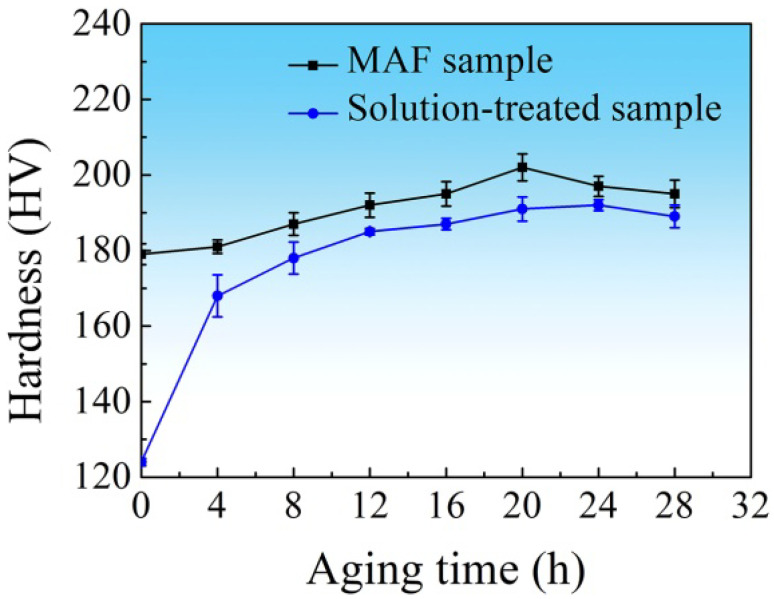
Hardness variations of the MAF and solution-treated samples with the time of aging.

**Figure 10:**
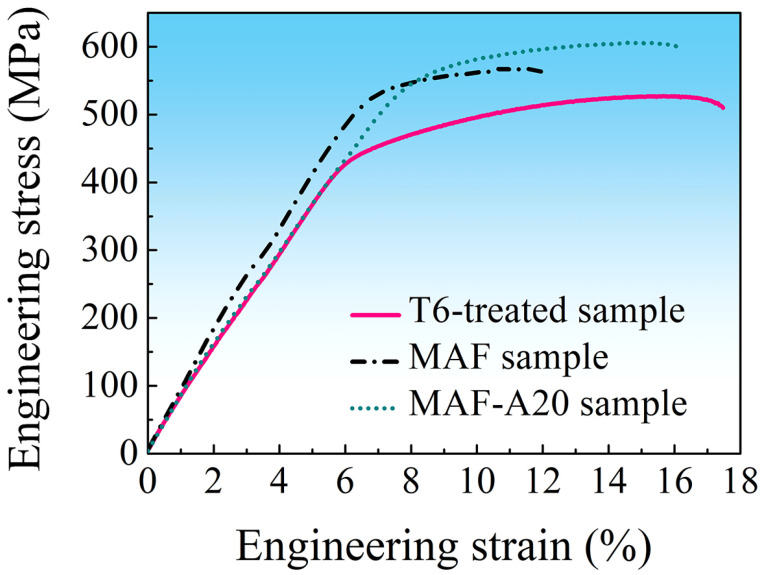
Engineering stress–strain curves of the T6-treated, MAF and MAF-A20 samples.

Figure 9 shows the hardness evolution of the solution-treated sample and the MAF sample during isothermal aging at 120 °C. After 24 h aging, the hardness of the solution-treated sample reached the highest value of 192 HV. However, after 20 h aging, the hardness of the MAF sample reached the highest value of 202 HV. Figure 10 shows the engineering stress–strain curves of samples. The T6-treated sample shows lower tensile strength but higher ductility (elongation to fracture). It is determined that the tensile strength and ductility are 527 MPa and 17.5%, respectively. As compared with the T6-treated sample, the MAF sample has high tensile strength but relatively low ductility of 567 MPa and 12.0%, respectively. However, the MAF-A20 processing results in the highest tensile strength, without much sacrificing of ductility. This shows a tensile strength of 606 MPa and an appreciable ductility of 16.2%. As compared with the T6-treated sample, the strength of MAF-A20 sample increases by 15%, and the ductility decreases by about 7.4%.

**Figure 11:**
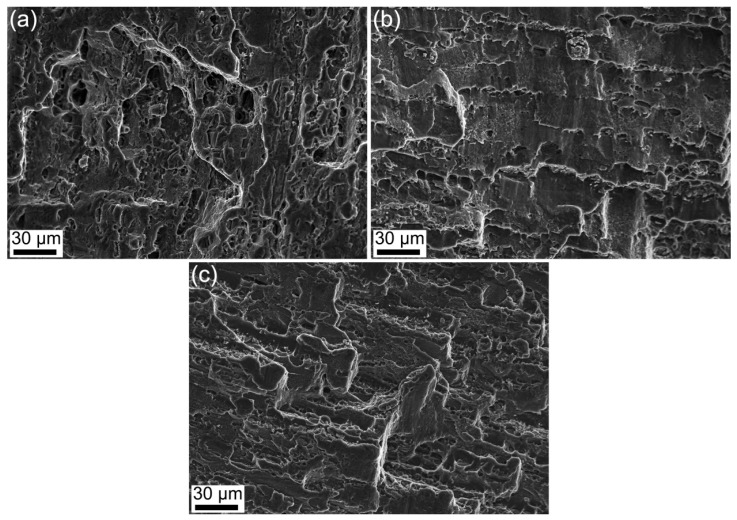
SEM images of tensile fracture morphology of samples: (**a**) T6-treated, (**b**) MAF, and (**c**) MAF-A20.

Figure 11 shows the tensile fracture morphology of the samples. As shown in Figure 11a, it can be easily seen that there are intergranular cracks and certain large and deep dimples in the fracture surface of the T6-treated sample, which is a typical combination of trans-granular failure and inter-granular failure. As shown in Figure 11b, it can be easily found that there are some cleavage steps and small dimples in the fracture surface of the MAF sample, which is a quasi-cleavage fracture. As shown in Figure 11c, it can be easily found that the fracture morphology of the MAF-A20 sample is similar to that of the T6-treated sample. Moreover, the tear ridges are more obvious. However, the dimple size is smaller than that of the T6-treated sample and there are some second phase particles at the bottom of the dimple. The fracture of the MAF-A20 sample is also a combination of trans-granular failure and inter-granular failure, but the ductility is slightly lower than that of the T6-treated sample.

In practice, the most important mechanical properties of materials, such as hardness, strength and ductility, are determined by the microstructure. In addition, the plastic deformation of polycrystalline materials is often associated with dislocation slip. The grain refinement, the dislocations, the precipitates and the solid solution may all contribute to the strength of ultrafine grained Al-Zn-Mg-Cu alloy [28]. During MAF processing, the SPD could impart severe strains with a high strain rate to the sample, which results in increasing dislocation density and decreasing grain size. Therefore, the strengthening mechanism of the MAF processing results from dislocation strengthening and grain refinement strengthening. Precipitation strengthening further improves the strength of the MAF sample during aging. After aging, residual stress reduction and dispersive precipitates within fine grains result in SPD and aging samples with higher ductility [17,18]. Due to a large number of dislocations and residual stress in the MAF-A20 sample, its ductility is still inferior to that of the T6-treated sample.

The essence of aging is to increase the diffusion kinetic energy of particles so that the solute dissolved in the supersaturated solid solution can be precipitated. Generally, the process of precipitation depending on the concentration fluctuation of alloy elements is considered as homogeneous nucleation [29]. Homogeneous nucleation needs thermal activation above a high energy barrier.

After MAF processing, a large number of new dislocations are produced. As heterogeneous nucleation sites, these high density dislocations can effectively reduce the nuclear barrier and promote precipitate nucleation [19,30]. In addition, high density dislocations can also act as diffusion channels to promote the rapid diffusion of alloying elements, and then promote the growth of the precipitated phase in the subsequent aging process [31]. This is consistent with the aging precipitation behavior of Al-Zn-Mg-Cu alloys that are processed by other pre-strain techniques [32,33,34]. Moreover, a large number of new dislocations are produced by the MAF processing. Besides showing in grains, some dislocations gather around the grain boundaries. The solute segregation at the grain boundaries is reported in an ultrafine grained Al-Zn-Mg-Cu alloy processed by an equal-channel angular pressing process [35] or high-pressure torsion [36]. In fact, both dislocation and grain boundary contribute to the nucleation, growth and coarsening of precipitates. Therefore, the precipitates on the grain boundary are coarse and discontinuously distributed in the MAF-A20 sample.

**Figure 12:**
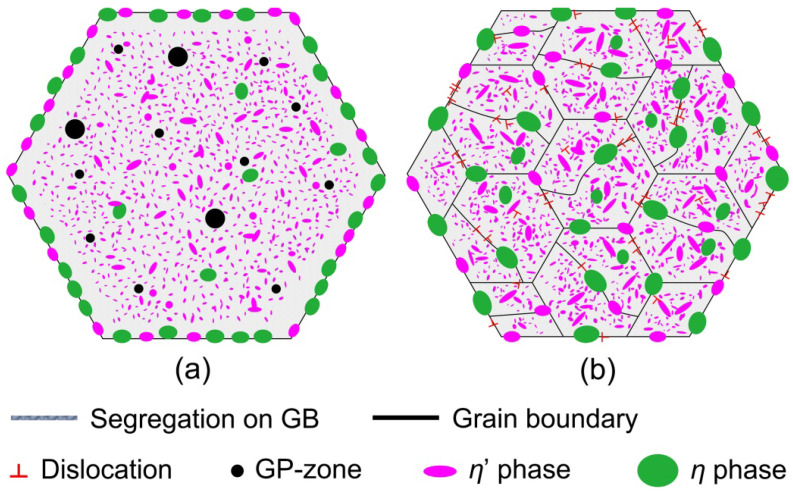
Schematic of microstructure of samples: (**a**) T6-treated; (**b**) MAF-A20.

Based on the above experimental information, the schematic of the microstructure of different samples can be shown in Figure 12. The microstructure of the T6-treated sample is composed of high density, homogeneously distributed small intra-granular η’ phases, a small number of larger inter/intra-granular η phases and some GP-zones, as shown in Figure 12a. Some researchers have suggested that the aging precipitation sequence after pre-deformation is the same as that of the usual precipitation sequence [19,37]. Since these dislocations accelerate the nucleation and growth of the precipitated phases, the microstructure of the MAF-A20 sample contains more intra-granular η’ phases, while a small proportion of the η’ phases have been grown or transformed into η phases, compared with the T6-treated sample, as shown in Figure 12b. Because the GP-zones almost transform into precipitated phases, the GP-zones can hardly be found in the MAF-A20 sample. Han et al. [5] also found that dislocations introduced by pre-deformation accelerate the movement of alloy elements towards grain boundaries, promoting the coarsening of grain boundary precipitates.

Moreover, the MAF sample achieves higher hardness in a relatively shorter aging time compared with the solution-treated sample. The hardness of the solution-treated sample is increased more sharply with aging, as shown in Figure 11. The reason is that recovery and grain growth will occur in the MAF sample during aging, which causes the hardness to increase slowly.

## 4. Conclusions

In this work, the effect of MAF and aging treatment on microstructure and mechanical properties of AA7075 aluminium alloy was researched. From the results of this work, the main conclusions can be summarized as follows:

(1) After MAF and aging, the alloy shows higher strength and ductility. Its strength is 15% higher than that of the T6-treated sample, while its elongation is 7% lower. The strengthening mechanism is due to dislocation strengthening, grain refinement strengthening and precipitation strengthening. The alloy with higher ductility results from reducing residual stress and dispersing precipitates within fine grains.
(2) After MAF processing, a high density of dislocations is produced, resulting in significant acceleration of the nucleation and growth of precipitated phases. The GP-zones almost transform into precipitated phases in the subsequent aging. Both dislocation and grain boundary are beneficial to the nucleation, growth and coarsening of the precipitates, which result in those on the grain boundaries being coarse and discontinuously distributed.

## Figures and Tables

**Table 1:** Preparation technology used for test samples.

Samples	Preparation Technology
Solution-treated	470 °C/2 h + water quench
T6-treated	470 °C/2 h + water quench + 120 °C/24 h
MAF	470 °C/2 h + water quench + multiaxial forging
MAF-A20	470 °C/2 h + water quench + multiaxial forging + 120 °C/20 h

**Table 2:** Calculation results of dislocation density.

Samples	*b*/nm	*d*/nm	<*e*^2^>/× 10^−6^	*ρ*/× 10^14^ m^−2^
MAF	0.286	33.35	4.17	7.4
MAF-A20		55.22	3.62	4.2

## Data Availability

All data included in this study are available upon request by contact with the corresponding author.
